# Integration with 3D Visualization and IoT-Based Sensors for Real-Time Structural Health Monitoring

**DOI:** 10.3390/s21216988

**Published:** 2021-10-21

**Authors:** Hung-Fu Chang, Mohammad Shokrolah Shirazi

**Affiliations:** R. B. Annis School of Engineering, University of Indianapolis, Indianapolis, IN 46227, USA; shirazim@uindy.edu

**Keywords:** structural health monitoring, Internet of Things, 3D modeling, real-time system

## Abstract

Real-time monitoring on displacement and acceleration of a structure provides vital information for people in different applications such as active control and damage warning systems. Recent developments of the Internet of Things (IoT) and client-side web technologies enable a wireless microcontroller board with sensors to process structural-related data in real-time and to interact with servers so that end-users can view the final processed results of the servers through a browser in a computer or a mobile phone. Unlike traditional structural health monitoring (SHM) systems that deliver warnings based on peak acceleration of earthquake, we built a real-time SHM system that converts raw sensor results into movements and rotations on the monitored structure’s three-dimensional (3D) model. This unique approach displays the overall structural dynamic movements directly from measured displacement data, rather than using force analysis, such as finite element analysis, to predict the displacement statically. As an application to our research outcomes, patterns of movements related to its structure type can be collected for further cross-validating the results derived from the traditional stress-strain analysis. In this work, we overcome several challenges that exist in displaying the 3D effects in real-time. From our proposed algorithm that converts the global displacements into element’s local movements, our system can calculate each element’s (e.g., column’s, beam’s, and floor’s) rotation and displacement at its local coordinate while the sensor’s monitoring result only provides displacements at the global coordinate. While we consider minimizing the overall sensor usage costs and displaying the essential 3D movements at the same time, a sensor deployment method is suggested. To achieve the need of processing the enormous amount of sensor data in real-time, we designed a novel structure for saving sensor data, where relationships among multiple sensor devices and sensor’s spatial and unique identifier can be presented. Moreover, we built a sensor device that can send the monitoring data via wireless network to the local server or cloud so that the SHM web can integrate what we develop altogether to show the real-time 3D movements. In this paper, a 3D model is created according to a two-story structure to demonstrate the SHM system functionality and validate our proposed algorithm.

## 1. Introduction

Important civil structures, such as bridges, energy utilities, nuclear power plants, and dams, require regular monitoring of their behaviors to support management decisions, reduce the loss of lives under natural disasters. Moreover, as a result of monitoring behaviors, necessary maintenance can be handled in time to minimize hazardous impacts on a large number of resources that are commonly employed within the constructions [1]. Structural health monitoring (SHM) aims to develop a system that can continuously check a structure, deliver current structural responses, and even alert when the structure exceeds the design limit. A structure’s behaviors often vary according to its age, usage, or environmental factors. SHM systems in the past research are designed with pre-assigned or specific types of sensor deployments due to the pre-defined calculation or in-device computations. Some SHM studies examined the monitoring approaches by evaluating the result afterward without investigating the possible application in real-time. However, when a natural disaster happens (e.g., earthquakes or gusty wind), an immediate reaction according to the observation on structural behaviors is critical. These rapid responses rely on real-time structural monitoring. Hence, a more effective SHM should intuitively visualize the structural real-time behaviors from the monitoring results across platforms and equip the flexibilities to adjust the computation with responding to the change in the sensor deployment, such as adjusting the number of devices or adapting the sensor location or sensor replacement. With the advancement of the micro-electro mechanical system (MEMS), wireless and computing technologies, combining sensors, micro-controller boards, client-side scripts, and internet servers to build a real-time cross-platform access-anywhere SHM system becomes more feasible.

A typical SHM system includes sensors, data processing components, and health evaluation units [2]. Recent technology development in these three areas has been shifted to utilize the latest standards and software libraries. For example, In the area of sensors or sensor networks, the traditional wireless sensor network within a local and close network (i.e., intranet) has been moved to the internet [3]. Various sensors attached to the microcontroller board, called the Internet of Things (IoT), can be easily programmed to interact with the servers on the internet. This new wireless sensor network based on the Internet of Things often involves web and database servers or even more complex computing infrastructure, such as the edge or cloud. The communication between IoT devices and servers often uses standard communication protocols, such as Hypertext Transfer Protocol (HTTP) or Message Queue Telemetry Transport (MQTT), to interact with each other and to communicate server systems across platforms over the internet [4,5]. IoT devices are referred to smart objects or things since they commonly offer a webpage-like user interface, which is implemented during the development phase, for their users to interact with them via mobile applications or internet browsers. These new wired and wireless sensor devices can also cooperate with multiple devices and different servers, unlike those in the past SHM research, which only focused on the computation and warning mechanism of a single device [6,7,8,9]. Instead of studying the monitoring and warning mechanism on a single IoT device, we would like to further include the consideration of the complex interactions among sensor devices, servers on the internet, and the user’s clients in the SHM system. Then, the SHM system migrates from a single server or personal computer within a local network to one that can integrate local computing servers, edge computing nodes, and various types of servers distributed across multiple regions on the cloud. At the same time, the design and development of this new SHM system also bring many challenges to node-to-node communication, data processing, algorithm, sensor deployment, and system architecture.

To show visualized structural behaviors and movements that can be used in design or study in the future, we built up a sensor device similar to other IoT smart things to collect monitoring data in our SHM system development. Our sensor device embeds a website-like user interface that can allow users to directly access the device and configure its device-server connection, deployment location at global coordinate, and sampling rate. Then, every sensor delivers real-time three-dimensional linear acceleration which will be translated to the displacement at global coordinate by the server in real-time. To display the actual movement of each part of the structure in a 3D model, the global displacement data at each sensor location need to be converted to each structural element’s displacement and rotation at its local coordinate. To incorporate a plug-and-play feature for sensor devices in our SHM, we create a key-value data format for keeping a generic element-to-element relationship. An algorithm is invented for handling this conversion. To process the enormous amount of data generated by the devices, we design a system architecture that can deal with this in real-time. This paper is an initial study, which continues our previous work related to IoT sensor research, toward our ultimate goal: a real-time SHM system that handles big data and heterogeneous sensors in an edge-cloud computing environment.

## 2. Related Works

Earlier SHM research studied a method or a sensor device to issue an early warning when the external force exceeded the design capacity of the structure or the implementation of the closed sensor network for monitoring [2,3,10,11,12,13,14,15]. Recent works focused on structural damage detections. Aloisio et al. [16] conducted a variance-based sensitivity analysis of various damage indicators to understand their possible usage limits. Zhoi and Wahab [17] incorporated the cosine measure and modal assurance criterion (MAC) indicators in the application of transmissibility-based structural damage detection. Sony and Sadhu [18] used various numerical simulations to validate the synchrosqueezing transform (SST) for progressive damage assessment. In applying IoT in the SHM, Muttillo et al. [19] built an IoT sensor system that integrated ADXL355 sensors, a Same3X8E ARM Cortex-M3 microcontroller, and a personal computer via a RS485 communication bus. Their suggested system evaluated the damage indicator but does not send monitoring data wirelessly to a server on the internet. Abdelgawad and Yelamarthi [20] used a USB Wi-Fi module, a Raspberry Pi 2 board, buffers, ADCs, DACs, and PZTs to build an IoT platform. The results will be sent to the server hosted in ThingWorx. Martinez et al. [21] created an IoT-based prototype that uses a Raspberry Pi 3 board, a USB wireless card, and accelerometer sensors to show the acceleration values in a x-y chart on a server. Aba et al. [22] developed an IoT-based sensor device that could monitor petroleum pipelines and showed the results in real-time on the ThingSpeak website.

## 3. Materials and Methods

### 3.1. Sensor Device

**Figure 1:**
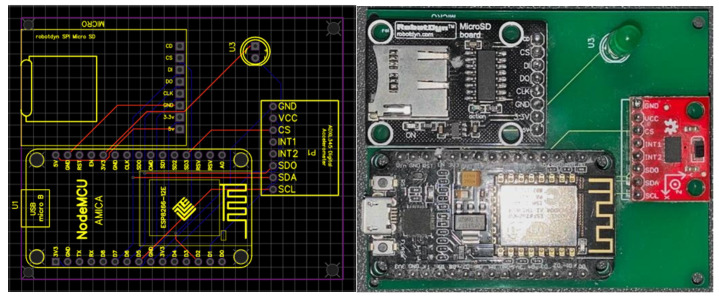
Schematic design and circuit board of the sensor device.

Our design choice emphasizes a device that can easily be installed in the existing structure and built with inexpensive costs. Hence, we selected the NodeMCU (a microcontroller board with a 32-bit ESP8266 chip), the ADXL345 accelerometer sensor [23], and a MicroSD board (see Figure 1). This integration almost occupied all the general purpose input/output (GPIOs) that NodeMCU provides (i.e., ten 3.3 voltage digital GPIOs and one 1.8 voltage analog input GPIO). In addition to the inexpensive cost for accelerometer sensors, the ADXL345 provides a better detection range and preciseness among MMA7455, MMA8451, MPU6060, MMA7660, and ADXL335 chips. The micro-SD board is not essential but it saves the monitoring results locally as backup, and the backup data can be analyzed offline if needed.

The wireless capability is provided by the ESP8266 chip. A small amount of data can be saved in its flash memory. The ESP8266 has a station infrastructure (STA) mode that sets a service set identifier (SSID) to allow other computers to connect to its wireless network; this means that the device links to the wireless network and obtains a local IP address during STA mode. Inside the device, we stored several web pages in the ESP8266 chip‘s built-in flash memory, letting users save configuration variables inside the flash memory. Because the user interface is a web page, the users can use a browser to set the key variables (e.g., the wireless network’s SSID, account and password, server host, etc.). During the initialization stage of the device, a universal unique identifier (UUID) is generated and saved in the flash memory. The server uses this UUID to associate with its deployed location, the relation with other devices, and the moving point in the 3D model. When real-time monitoring records associated with the device’s identification number are received by the server, the overall movement of the 3D structural model is calculated according to our algorithm and shown to the client.

It is critical to ensure sufficient power to a sensor device to monitor the structure in real-time. The micro USB port in the NodeMCU provides the entire device power. Using the micro USB port extends the sensor device’s power source options (e.g., using a replaceable high-capacity battery) and increases the device’s portability. On the other hand, the device buffers the monitoring data in the built-in flash memory and then pushes the data via the network to an internet server. All the computations are performed in the server. Decreasing the number of data transmission times to the server and shifting the computation from the sensor device to the server should reduce the power consumption.

### 3.2. System Architecture

**Figure 2:**
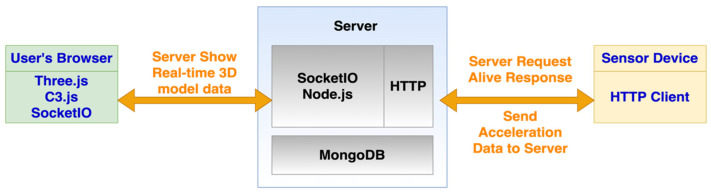
Architecture of the SHM system.

Modern software widely applies various web technologies to facilitate inter-operability between programs or to exchange data among servers. Due to the recent advance of Javascript technology, particularly in the server-side run-time environment (i.e., Node.js) and client-side framework and libraries, software can enjoy the benefits from the Javascript; that is, its scalability, high-performance, and asynchronous non-blocking executions. Hence, our server software integrates various Javascript server-side and user interface libraries—Node.js, SocketIO, Three.js, and C3.js (see Figure 2). All the server-side Javascript components run on the Node.js environment. The SocketIO is for real-time communication, which sends bi-directional messages according to the pre-defined events, between the client side (i.e., user’s browser) and the server side. So, each axis’ displacement values as well as the transformation and movement data of each 3D element are embedded in the server-to-client message sent via the protocol created by the SocketIO on both sides. The Three.js on the client-side page uses WebGL to provide application programming interfaces (APIs) for users to create and render the 3D model. We create an online 3D model editor and a real-time display based on the Three.js. Since Three.js can run on any browser for online 3D model rendering and editing, the 3D model can be displayed in any client [24]. The C3.js is the chart library to show real-time acceleration values on the X-Y line chart.

The software architecture of our server is layer style. The upmost layer is HTTP, responsible for the request and response between sensor device and server. All the IoT sensor device monitoring results are pushed to this server according to its sampling frequency via HTTP protocol. All the required computations are in between the HTTP and data storage layers. Inside the computation part, each monitoring point’s global displacements are calculated from monitored acceleration values and then the global displacements can be converted into each 3D structural element’s rotation and shift. Regarding the data storage, the system uses MongoDB, which is classified as a document-oriented (NoSQL) database.

**Figure 3:**
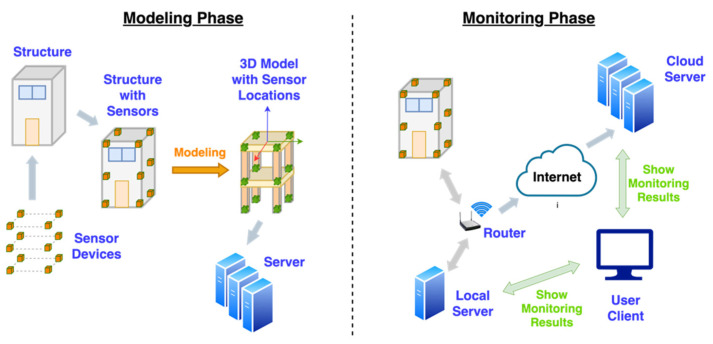
Two different types of the SHM usages.

There will be two different kinds of usages of the SHM system (see Figure 3). Firstly, the 3D model is formed according to the real-world civil structure and then all sensor device details (e.g., locations and identification number) are scaled down to the model. All the model and sensor details are saved in the database. When the monitoring starts, all real-time monitoring results collected from IoT sensor devices are sent to the server via the wireless network. To reduce the transmission data, the monitoring results are firstly sent to the local server. The local server then sends replica and computation results to the cloud server. During this monitoring phase, once the user’s client accesses any server, the user can see the 3D model moving in real-time.

### 3.3. Three-Dimensional Structural Model Visualization

#### 3.3.1. Challenges and Assumptions

The real-world structural displacement combines the deformation of each element (e.g., bending or shrinking) and the ground movement. The actual deformation of the structure depends on its material, shape, and force. After the structure is built, the composition of the material is often unknown. That is why most SHM sensor devices only monitor acceleration and issues warnings when the monitoring acceleration is close to the designed value. The nature of having many unknown factors of the to-be-monitor after-built structure particularly brings tough challenges to our system for displaying each structural element’s actual deformation and displacement on its 3D model according to three-dimensional monitored displacements.

The overall displacements derived by the sensor’s monitoring results are the combinational effects of horizontal and vertical forces. Traditional systems use forces and material stress and strain model to derive the displacements of the structural element. One assumption has to be made to treat the monitored displacements as an aftereffect of complex combinational forces and only use displacements to illustrate the structure’s 3D model movement. We assume that every column section between two monitoring points is a rigid body—no deformation. Therefore, the assumption overlooks the initial linear and later non-linear behaviors of most construction materials. We attempt to overcome this weakness by putting more sensors on one column. If four corners of a floor are monitoring points that are supported by columns, the deformation of the floor can be decided by the displacements of the column at each corner.

Our primary focus is on horizontal displacements. We limit our problem to horizontal measurements, which implies that the vertical (i.e., *z*-axis) measurement is neglected and is regarded as a shift to the entire structure. Because horizontal forces mainly cause a structure’s horizontal displacement, the contribution of the vertical force on the horizontal displacement can be neglected.

#### 3.3.2. Structural Global Displacement

The displacement can be derived from the numerical integration of the acceleration. In other words, the area under the graph of the velocity curve over time is the displacement. Previous studies [25,26,27,28] have suggested methods to calculate it from the accelerometer so we applied those integration equations to get the global displacement at each sensor location.

Getting global displacement at each monitoring point is the first step to visualize the 3D model movements. However, the global displacement is insufficient to understand how each connected structural element moves (e.g., how floor and column moves) because elements in the 3D model rotate and move at their local coordinate. As a result, we must figure out a way to translate the global displacements into each element’s rotation and displacement at its local coordination system.

#### 3.3.3. Conversion from Global Displacement to Element’s Local Rotation and Displacement

**Figure 4:**
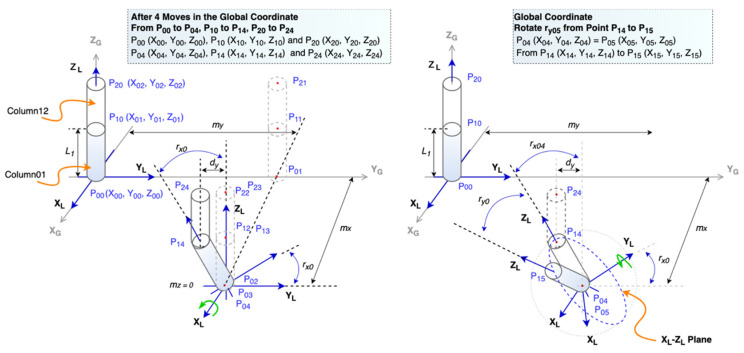
Movements caused by X-Y forces.

Earthquakes produce three-dimensional ground forces. The displacements and rotations of the structure can be viewed as the result of these three ground forces. Figure 4 shows that the movements of the two connected columns (i.e., Column01 and Column12) are caused by the horizontal forces from the position at time *t*_0_ to another one at *t*_1_. Every P_ij_ point is a sensor monitoring location, and signals the point at the *i*-th position at the j-th step. To illustrate how to derive global-to-local conversion equations under this situation, we divide the movement between these two timestamps into six steps. The first three steps are displacements along with the Y_G_, X_G_, and Z_G_ axes, and the latter three steps are rotations at X_L_, Y_L_, and Z_L_ axis. Since all six steps are caused by forces along with X_G_ and Y_G_, there is not any rotation at Z_L_ axis.

**Figure 5:**
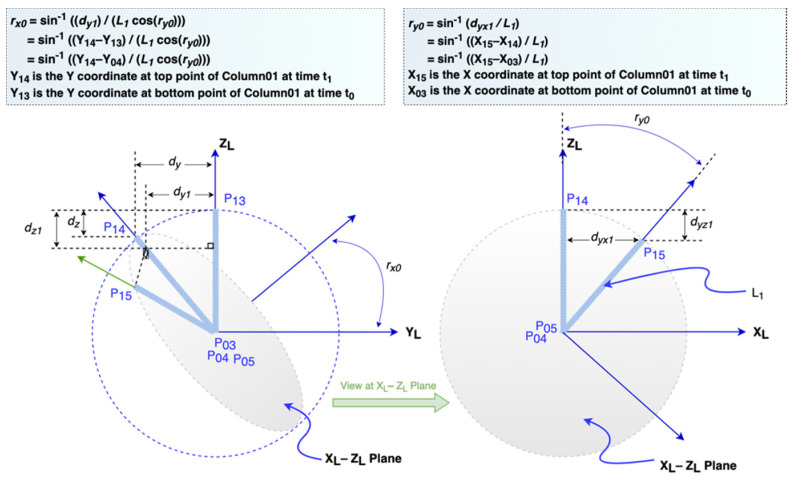
The rotation *r_x_*_0_ and *r_y_*_0_ at the Column01.

From Figure 4 and Figure 5, we can derive Equations (1) and (2) where *L*_1_ is the length of the Column01, *X*_1_(*t*_1_) and *Y*_1_(*t*_1_) are coordinates of the top point of the Column01 at time *t*_1_, *X*_0_ (*t*_1_) and *Y*_0_ (*t*_1_) are coordinates of the bottom point at Column01 at time *t*_1_, *r_y_*_0_ is the angle at Y_L_ axis, and *r_x_*_0_ is the angle at X_L_ axis.

(1) $$r_{y0}=\left(\left(X_{1}\left(t_{1}\right)–X_{0}\left(t_{1}\right)\right)/L_{1}\right)\;$$

(2) $$r_{x0}=\left(\left(Y_{1}\left(t_{1}\right)–Y_{0}\left(t_{1}\right)\right)/(L_{1}cos\;cos\;r_{y0}\right))$$

Both rotations will also contribute *z*-axis displacement *d_z_*_1_. The value at time *t*_1_ can be established after *r_y_*_0_ and *r_x_*_0_ are computed.

(3) $$d_{z1}=L_{1}\left(1-cos\;cos\;r_{y0}\;cos\;cos\;r_{x0}\right)$$

(4) $$Z_{1}\left(t_{1}\right)=L_{1}cos\;cos\;r_{y0}\;cos\;cos\;r_{x0}$$

When we have multiple connected columns and two rotations are applied at a particular column *n*, its two rotation angles and *z*-axis displacement can be calculated from the generalized equations as follows:

(5) $$r_{yn-1}=\left(\left(X_{n}\left(t_{i}\right)–X_{n-1}\left(t_{i}\right)\right)/L_{n}\right)$$

(6) $$r_{xn-1}=\left(\left(Y_{n}\left(t_{i}\right)–Y_{n-1}\left(t_{i}\right)\right)/(L_{n}cos\;cos\;r_{yn-1}\right))$$

(7) $$d_{zn}=L_{n}{\left(1-cos\;cos\;r_{yn-1}\;cos\;cos\;r_{xn-1}\right)}_{\;}$$

(8) $$Z_{n}\left(t_{i}\right)=L_{n}cos\;cos\;r_{yn-1}\;cos\;cos\;r_{xn-1}$$

where *L_n_* is the length of the n-th column, *X_n_*(*t_i_*) and *Y_n_*(*t_i_*) are coordinates of the top point of the *n*-th column at time *t*_1_, *X_n_*_−1_(*t_i_*) and *Y_n_*_−1_(*t_i_*) are coordinates of the bottom point of the *n*-th column at time *t_i_*, *r_yn−1_* is the angle at Y_L_ axis, and *r_xn−1_* is the angle at X_L_ axis.

**Figure 6:**
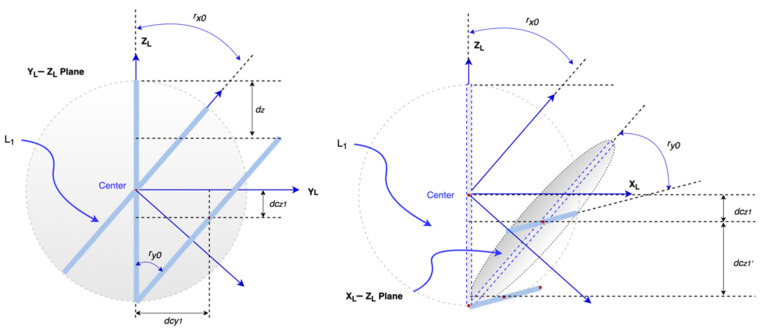
Shifts after two rotations.

However, 3D models are designed to rotate at the center of the object, unlike the above scenario—rotating at the bottom of each column. To translate the rotation in Figure 5 to the rotation at the bottom, the 3D model element must include a shift after it rotates at the same required angle. Given that there are multiple columns connected, at the *n*-th column, we have to consider two types of shifts. The first one is from the displacement at its bottom point, which is caused by the (*n*−1)-th element’s shift. The bottom point’s shift is measured at time *t_i_* to *t_i_*_−1_ and its movement is in three directions so each direction’s displacement can be calculated by the difference of coordinate value at each axis. Therefore, the bottom point displacement at the *x*-axis is *X_n_*_−1_(*t_i_*) − *X_n_*_−1_(*t_i−_*_1_) and the same principle can be applied to the *y* and *z*-axis. The second type of movement caused by the rotation is illustrated in Figure 6. For the *n*-th column rotating around the *x* and *y*-axis, the total displacements are shown as follows:

(9) $$d_{cyn-total}=(L_{n}/2)\left(1-sin\;sin\;r_{xn-1}\right)+\left(Y_{n-1}\left(t_{i}\right)–Y_{n-1}\left(t_{i-1}\right)\right)$$

(10) $$d_{cxn-total}=(L_{n}/2)\left(1-sin\;sin\;r_{yn-1}\right)+{\left(X_{n-1}\left(t_{i}\right)–X_{n-1}\left(t_{i-1}\right)\right)}_{\;}$$

The displacement at the *z*-axis is the sum of the center point declining introduced by both rotations (i.e., *d_czn_* + *d_czn_’*) and also the bottom point’s movement.

(11) $$\begin{array}{ll} d_{cyn-total} & =d_{czn}+d_{czn}^{\prime }\\ & =(L_{n}/2)\left(\left(1-cos\;cos\;r_{xn-1}\;\right)+\left(1-cos\;cos\;r_{yn-1}\right)\right)\\ & +\left(Z_{n-1}\left(t_{i}\right)–Z_{n-1}\left(t_{i-1}\right)\right) \end{array}$$

The global coordinate of the center point measured at the time *t_i_* is in the following equation.

(12) $$\left(X,\;Y,\;Z\right)=\left(\frac{X_{n}\left(t_{i}\right)+X_{n-1}\left(t_{i}\right)}{2},\frac{Y_{n}\left(t_{i}\right)+Y_{n-1}\left(t_{i}\right)}{2},\frac{Z_{n}\left(t_{i}\right)+Z_{n-1}\left(t_{i}\right)}{2}\right)$$

**Figure 7:**
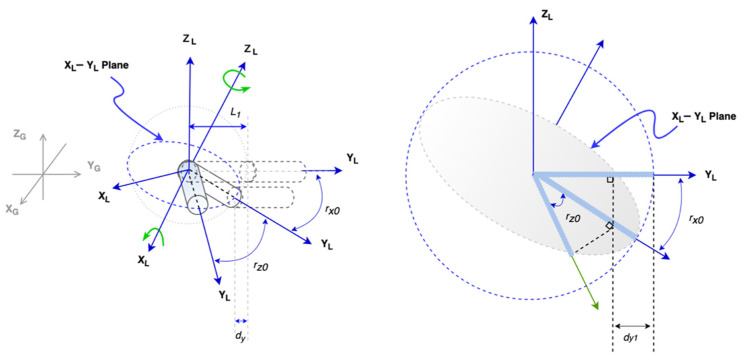
Two rotations of the beam.

We can apply the same idea to derivate the conversion from global displacement to the local beam element’s rotation and displacement. For a beam parallel to the *y*-axis, its rotations can be at the *z*- or *x*-axis (see Figure 7).

(13) $$r_{xn-1}=\left(\left(X_{n}\left(t_{i}\right)–X_{n-1}\left(t_{i}\right)\right)/L_{n}\right)$$

(14) $$r_{zn-1}=\left(\left(Z_{n}\left(t_{i}\right)–Z_{n-1}\left(t_{i}\right)\right)/(L_{n}cos\;cos\;r_{xn-1}\right))$$

(15) $$d_{yn}=L_{n}\left(1-cos\;cos\;r_{xn-1}\;cos\;cos\;r_{zn-1}\right)$$

Similarly, for a beam parallel to the *x*-axis, when its rotations are in the z- or *y*-axis, the equations for getting the angles are Equation (16) and Equation (14), respectively.

(16) $$r_{yn-1}=\left(\left(Y_{n}\left(t_{i}\right)–Y_{n-1}\left(t_{i}\right)\right)/L_{n}\right)\;$$

(17) $$d_{xn}=L_{n}\left(1-cos\;cos\;r_{yn-1}\;cos\;cos\;r_{zn-1}\right)$$

#### 3.3.4. Sensor Deployment

**Figure 8:**
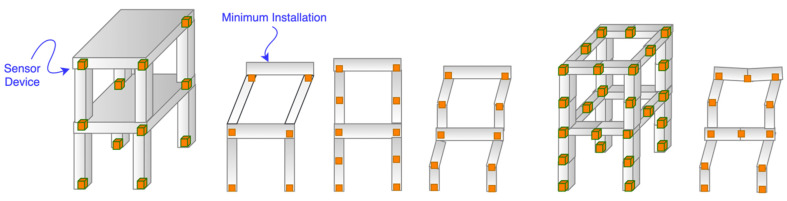
Sensor deployment.

Past research [29,30,31] already discussed the importance of IoT device deployment. In our system, the sensor location is particularly critical because it determines the details of the overall monitored structure’s transformation and movement. Sensors are installed at the monitoring points. The sections between two sensor devices are treated as connected columns or beams. As a result, when a structure is modeled, its model must compose of separated elements, where both ends of the element are the monitoring points. Adding sensors to columns and joints must happen before attaching them to beams. By adding more sensor devices to each structural element (i.e., beam or column), the non-linear deformation can be shown. Figure 8 shows that the more detailed deformation can be displayed while the number of sensors increases. We must know that the minimum sensor installations are at the joints on the columns. Otherwise, our above equations cannot be adequately applied for displaying movements in our SHM system.

#### 3.3.5. Timestamp

**Figure 9:**
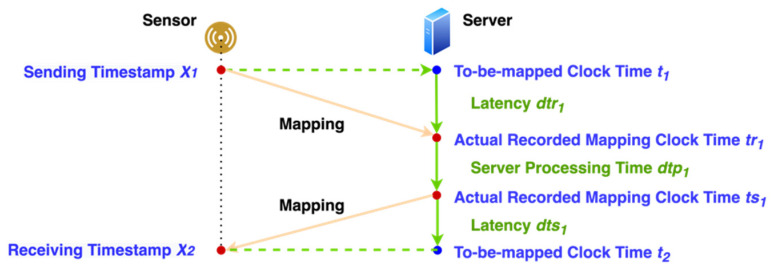
Mapping between sensor device local timestamp and server clock time.

Timestamp or time is critical to the real-time system. The sensor device only contains a timer that measures time in milliseconds starting from zero when it boots. The timestamp keeps increasing until it shuts down. Our way to get time is to map the timestamp from its timer to the server’s clock time by sending a request and receiving the server’s response for inquiry and recording the time. Figure 9 shows that the timestamp x_1_ should be mapped to the server’s clock time *t*_1_. Due to latency, when the server records the clock time *tr*_1_ associated with *x*_1_, the time already passed *dtr*_1_. Hence, the mapped server time cannot be accurate.

We try to calculate the average latency and use it to adjust the clock time so we can get the recorded mapping time closer to the one that should be mapped. The following equations show how we map timer *x*_1_ and *x*_2_ to clock time *t*_1_ and *t*_2_, respectively.

(18) $$Average\;Latency\;dt_{1}=((x_{2}-x_{1})-\left(ts_{1}-tr_{1}\right))/2$$

(19) $$x_{1}\to t_{1}≅tr_{1}-dt_{1}$$

(20) $$x_{2}\to t_{2}≅ts_{1}+dt_{1}$$

According to the equations above, mapped clock time in each sensor device is an approximate value. Therefore, it is not easy for all devices to get the acceleration at the same clock time. What is worse is that multiple devices receive different network latencies. So, the best strategy is to reduce the latency. As a result, having a local server to communicate with all the servers becomes a better solution.

#### 3.3.6. Data Format

To use our suggested method to display the overall movement of the structure in its 3D model, the SHM system needs to save more than measurement data. Sensor deployment information and relation between different types of structural elements, such as floors, columns, and beams, are also required. For example, to calculate the displacement of a column, all the monitoring points at both ends of the connected parts are needed.

**Figure 10:**
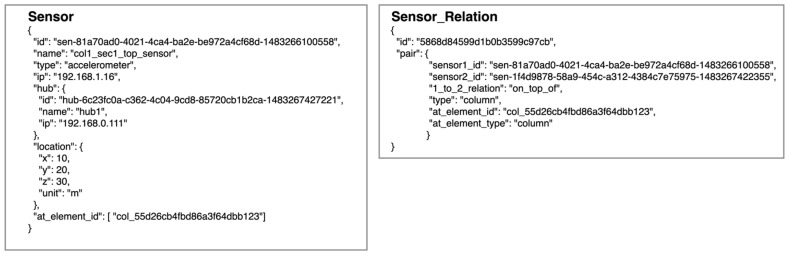
Example of sensor and sensor relation data.

All the relations are organized in key-value pairs and arrays in the JavaScript Object Notation (JSON) format. Figure 10 shows an example of how the data are saved.

The structural 3D model is created after the user edits it on the website. The model data saved in the JSON format contain camera information, geometries, materials, and objects. After the monitoring data are converted into each object’s rotation, displacement, or even transformation, the dynamic behaviors under external forces can be shown in its 3D model.

## 4. Implementation and Validation

### 4.1. Implementation

#### 4.1.1. Sensor Device Software

**Figure 11:**
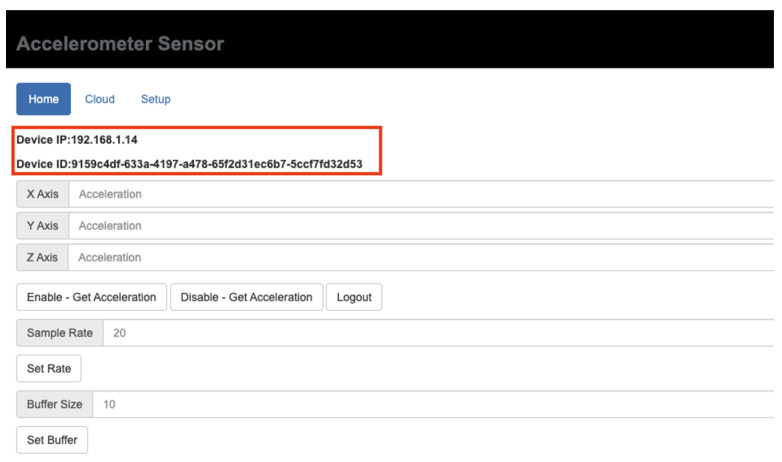
Home tab of sensor device user interface.

**Figure 12:**
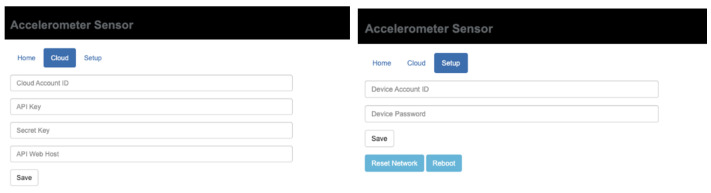
Cloud and setup tabs of sensor device user interface.

The sensor device’s user interface is a tab-like web page (see Figure 11). The “Setup” tab allows users to connect to a wireless network so that the device can acquire an IP address from the router (see Figure 12). Users configure the server IP and hostname in the “Cloud” Table After all the required information is set, the “Home” tab shows its IP and identification number. The “Home” tab provides the real-time acceleration measurements at x, y, and z directions after the enable button is clicked. Users can also select a different sample rate. The buffer size input is used to specify the internal memory buffer for caching the monitoring data. As for the “Setup” tab, user account and password can be set in order to protect illegal access to this user interface of this sensor box. In this way, saved configurations will not be altered.

#### 4.1.2. Server Software

The implementation contains three major parts: (1) project, structural model, and sensor information; (2) 3D model building tool; and (3) 3D model real-time movement display. Users can create a project that includes various structures. Sensor device locations and identifications are saved in the server. After the user creates a 3D model in our application, the model data will automatically be saved in the server for later display. The last part is the real-time view for 3D model movement. The user can move the camera position or zoom in/out the view to see the movement from different angles.

**Figure 13:**
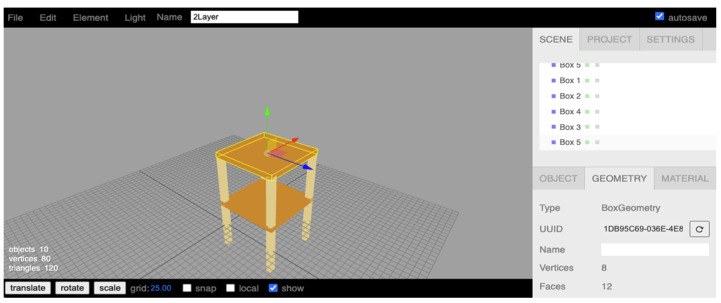
Two-story structure on 3D model editor of the SHM system.

**Figure 14:**
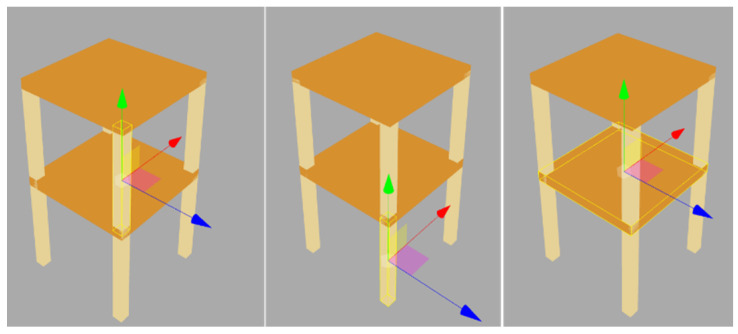
Local coordination at each element.

Figure 13 shows the 3D model editor in our server application inside our SHM system. All the structural elements are built by using the “box” object. The Three.js client-side component can allow users to use any browser to edit their 3D models according to the to-be monitored structure. Because of using a browser, the feature of editing anywhere is realized. We also want to emphasize why global to local conversion is necessary in real-time display. In Figure 14, the local coordinate on each structural element is at its center. It is important to note that the displacement, rotation, and transformation are all at their local coordination system instead of the global coordinate system. Each element’s local coordination system will change after its local rotation or shift. It introduces the challenges of global to local conversion.

### 4.2. Validation

Our validation targets correctly display real-time 3D movements according to the structural monitoring data sent by multiple sensor devices. Unsynchronized monitoring timestamps across different sensor devices or inaccurate global and local conversion equations will cause the detached structural model parts (e.g., a beam disconnects with a column) or unreasonable movement of elements (e.g., a clockwise rotation becomes an incorrect counterclockwise rotation).

**Figure 15:**
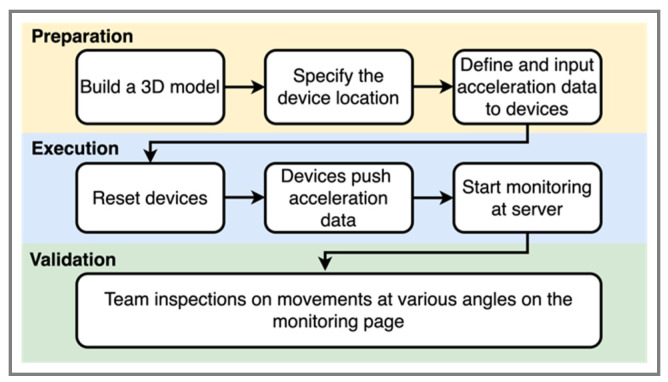
Global to local conversion validation procedure.

There are two parts in the validation. First, we test the time adjustment in the sensor device. Four devices are put on the small-scale shaking table and shook with a maximum acceleration of 0.2 g. The monitored acceleration values will be examined at each timestamp. The second part is a simulation for investigating our conversion approach, which is a step before we later conduct a scaled structure experiment on a shaking table. We build a two-story 3D model for displaying the movement. On the model, the locations of the sensors are at each joint so we use eight sensor devices to send the simulation data to the server on the local network. The movements of the 3D model will be checked via a browser that accesses the server. Figure 15 shows the procedure of how we deal with the conversion equation validation.

#### 4.2.1. Time and Latency

**Figure 16:**
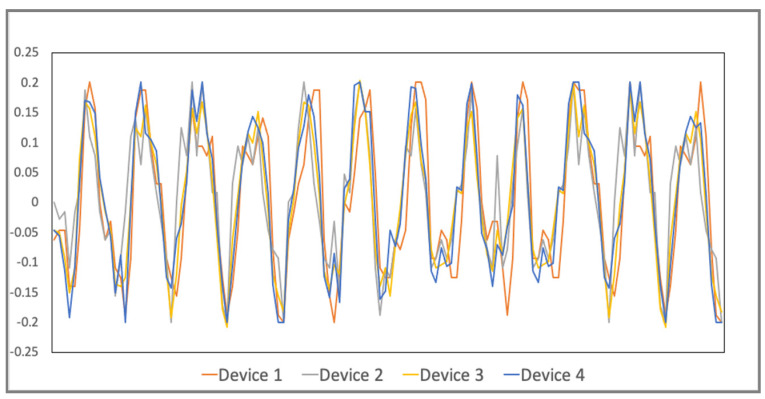
Acceleration monitoring of four sensor devices.

Since four devices are moved at the same acceleration, after our time adjustment for each device, all of them are expected to get the same acceleration value at the same timestamp. This implies that their wave pattern should be very close to each other. According to Figure 16, the time adjustment is accurate because it shows four monitoring curves are very close. Table 1 shows the standard deviation of the acceleration values of the four devices at each timestamp. A very low maximum standard deviation also validates our time adjustment method.

#### 4.2.2. 3D Movement Calculation

Our server for hosting the real-time SHM system is implemented in the one core CPU, 512MB ram, and Ubuntu OS machine on the local network. The two-story structural 3D model (see Figure 13) is built to validate our proposed global to local conversion. Every sensor is assigned at each joint so eight devices are used in the simulation. The acceleration data pushed by the sensors continuously contribute the displacements at the sampling rate of 20 Hz to the server. It is noteworthy that the monitoring sample time interval is different from the display time interval. In our validation, we use one second. This can avoid a display lag on the client’s browser, which is caused by too frequent updates and heavy client-server data traffic.

**Figure 17:**
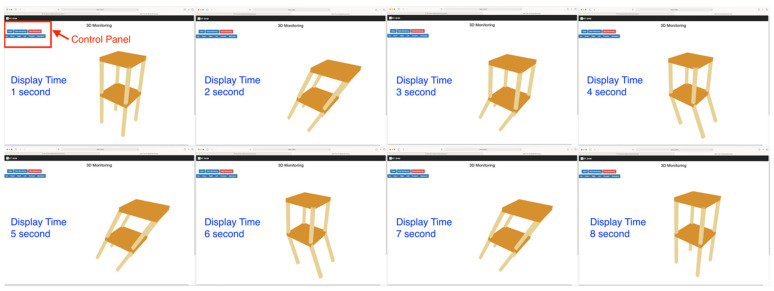
Two-story model movements.

Two criteria were used to validate the global to local conversation. At each joint, connected elements cannot detach during every movement because our conversion equations are under the assumption that there are no broken joints and rigid body rotations and displacements. The other criterion examines rotations or displacements in the correct direction. Three people inspected the movements by using their browser at the same time, checked the model movements from various angles by moving the camera, and then made observation notes for each movement. Figure 17 shows that eight movements repeat the same sequence continuously because the sensor devices keep delivering a series of pre-defined acceleration values. Throughout the whole simulation, no disjoints between elements or wrong rotations were seen by the inspectors. This indicated that the system displayed movements correctly and validated our conversion equations. Because a successful 3D model movement also relies on synchronous monitoring time and accurate movement interpolation, correct 3D model movements also indirectly validate our time synchronization and interpolation methods. These validations are performed through the simulation, which prepares our SHM system to reproduce the actual movements of the to-be-monitored structure.

## 5. Discussion and Conclusions

In this paper, we proposed a SHM system that can demonstrate real-time structural movements in a 3D model on the website so that users can view monitoring results by using any client device that has browsers. In our SHM system, the users can build up their structural 3D model according to the real-world structure and save all the sensor information. With the model and sensor data, once the real-world structure moves under the external force, the user can see the real-time movements on the display webpage.

Our validation results showed that our conversion method, monitoring time synchronization approach, and displacement adjustment equation could display the overall behaviors correctly. Our current result is the initial step for accurately displaying real-world structural monitoring results in a 3D model. That means, we have to consider elastic and non-linear behaviors of the structural element. We understand that our conversion equations have limitations due to our rigid body assumptions. Real-world structural behaviors under external forces do not merely act as a rigid body. Although we can use more sensors to display bent structural elements approximately, we will need to study a 3D deformation equation to show the non-linear behaviors of every structural element in the future. We also know that using acceleration values to get displacements might not be ideal. However, we argue that this deficiency does not impact our proposed approach. In other words, once we can apply a more accurate displacement measurement technique, our SHM can display more realistic 3D movements of the to-be monitored structure.

## Figures and Tables

**Table 1:** Standard deviation of accelerations at each timestamp.

Maximum Standard Deviation	Minimum Standard Deviation
0.0950045	0
